# Beyond the average: why data spread matters in clinical studies

**DOI:** 10.36416/1806-3756/e20250142

**Published:** 2025-05-21

**Authors:** María Teresa Politi, Juliana Carvalho Ferreira, Cecilia María Patino

**Affiliations:** 1. Methods in Epidemiologic, Clinical, and Operations Research - MECOR - program, American Thoracic Society/Asociación Latinoamericana del Tórax, Montevideo, Uruguay.; 2. Laboratorio de Estadística Aplicada a las Ciencias de la Salud (LEACS), Departamento de Toxicología y Farmacología, Facultad de Ciencias Médicas, Universidad de Buenos Aires, Buenos Aires, Argentina.; 3. Divisão de Pneumologia, Instituto do Coração, Hospital das Clínicas, Faculdade de Medicina, Universidade de São Paulo, São Paulo (SP) Brasil.; 4. Department of Preventive Medicine, Keck School of Medicine, University of Southern California, Los Angeles, CA, USA.

## PRACTICAL SCENARIO

A pulmonologist is conducting a quality improvement study to evaluate whether lung function, measured by FVC, differs across two outpatient asthma centers within the same health care system. Her research question is: “Do patients from Center A have better lung function than those from Center B?”

**Figure 1:**
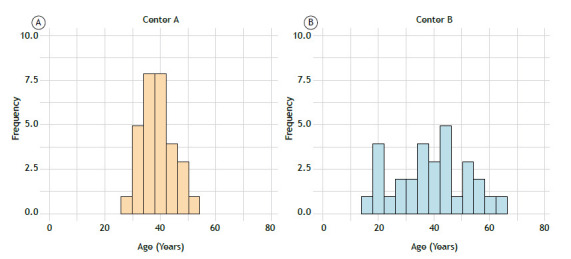
Age distribution of adult outpatients from respiratory consultations. In A, Center A, characterized by a narrow age dispersion. In B, Center B, characterized by a wide age dispersion. N = 30 patients per group.

Since spirometry values are strongly influenced by patient age, she first checks whether the two groups are comparable in terms of age distribution. The mean age in Centers A and B, respectively, is 39.2 years 38.9 years. At first glance, the groups appear similar, and she assumes that age is unlikely to affect the comparison of spirometry outcomes between the centers. However, upon further examination of the age histograms for each center, she finds that the age dispersion varies markedly between the groups (Figure 1). Should she reconsider her conclusion?

## LIMITATIONS OF THE MEAN AS A CENTRAL TENDENCY MEASURE

When summarizing clinical data that are continuous, we typically report a measure of central tendency-mean or median- and a measure of spread-standard deviation (SD) or interquartile range (IQR). These measures of spread are not just statistical formalities; they inform how we accurately interpret patient data that inform clinical decisions.

The mean is the average of a continuous variable (e.g*.,* age), and is calculated by dividing the sum of all values in a dataset by the total number of observations.^1^ It serves as a measure of central tendency by summarizing the distribution using a single value. However, the mean is sensitive to extreme values, which can distort its true value in study samples (accuracy) and make it misleading, especially when the distribution of the data is skewed or when outliers are present. For this reason, the mean is typically reported when data is normally distributed, where values are symmetrically spread around the mean, and outliers are less likely to impact the interpretation of results.

The median, on the other hand, is the middle value of a continuous variable in a dataset arranged in rank order. It divides the data into two halves: 50% of values fall below or equal to it, and 50% above or equal to it, placing it at the 50th percentile.^1^ When the number of observations is odd, the median is the value positioned exactly in the middle of the ordered dataset. If the number of observations is even, it is calculated by averaging the two central values. Unlike the mean, the median is a more robust measure because it is less influenced by extreme values, since it depends only on central data points rather than the entire distribution of the data. As such, the median is preferred for non-normally distributed data, where skewness or outliers of the data may make the mean less representative of the “typical” observation.^1^

Choosing between the mean and the median is more than a statistical decision, because it can influence the clinical interpretation of the data. If a small number of patients have very high or very low values on a variable, the median might reflect the “typical” patient more accurately.^2^

## SD: MEASURING SPREAD AROUND THE MEAN

The SD measures how much individual data points differ from the mean. It reflects the average distance of each value from the mean of the dataset.^2^ When data points are tightly spread around the mean, the SD is small; when they are more widely spread, the SD increases. In the extreme case when all values are identical, the SD is zero. Because it shares the same units as the mean, the SD is straightforward to interpret.^1^ In our practical scenario, the SD of age was 5.8 years for Center A and 13.0 years for Center B. This wide discrepancy suggests that while the mean ages are very similar, the two groups are not truly comparable. A broader spread in Center B may reflect greater patient diversity, requiring additional stratification in analysis.^1^

## IQR: CAPTURING THE MIDDLE 50%

The IQR complements the median and is commonly used for non-normally distributed data.^2^ It is calculated as the difference between the 25th and 75th percentiles, representing the range within which the central 50% of values lie. By excluding the lowest and highest quartiles, the IQR is resistant to the influence of outliers.^2^ In our example, the IQR regarding age was [36.3-42.8 years] and [30.5-49.0 years] for Centers A and B, respectively. Similarly to the SD, the IQR uses the same units as the original data, which enhances its interpretability.^1^

In our practical scenario, differences in age dispersion suggest that comparing spirometry values without adjusting for age distribution could be misleading. By adjusting for age, the pulmonologist accounts for the variability in the data to draw more accurate conclusions, reinforcing the importance of dispersion measures in clinical research.

## TAKE-HOME MESSAGES FOR CLINICIANS

Never report means or medians alone without a measure of spread.

Use the median and IQR for variables with skewed data or that include outliers.

Use the mean and SD for variables with symmetric, normally distributed data.

Using statistics to assess both central tendency and variability provides a more complete and clinically relevant picture.

In clinical research, knowing where the average lies is helpful, but knowing how far the rest of the data stray from it is what truly makes comparisons meaningful.
